# Pop goes the tumour! Spontaneous haemorrhage of a hepatocellular carcinoma tumour

**DOI:** 10.1093/jscr/rjaa598

**Published:** 2021-01-31

**Authors:** Nelson Agostinho, Harinder K Bains, Cameron P Douglas, James Walcott, Kilian G M Brown, Robert Rawson, Charbel Sandroussi, Meegodage Ruwan Suramal Perera

**Affiliations:** Department of General Surgery, Dubbo Base Hospital, Dubbo, NSW, Australia; Department of General Surgery, Dubbo Base Hospital, Dubbo, NSW, Australia; Department of General Surgery, Dubbo Base Hospital, Dubbo, NSW, Australia; Department of Upper Gastrointestinal and Hepatobiliary Surgery, Royal Prince Alfred Hospital, Camperdown, NSW, Australia; Department of Upper Gastrointestinal and Hepatobiliary Surgery, Royal Prince Alfred Hospital, Camperdown, NSW, Australia; Department of Tissue Pathology and Diagnostic Oncology, Royal Prince Alfred Hospital, Camperdown, NSW, Australia; Department of Upper Gastrointestinal and Hepatobiliary Surgery, Royal Prince Alfred Hospital, Camperdown, NSW, Australia; Department of General Surgery, Dubbo Base Hospital, Dubbo, NSW, Australia

**Keywords:** spontaneous haemorrhage, liver lesion, hepatobiliary, upper gastrointestinal surgery

## Abstract

An 84-year-old man presented to a rural hospital in Australia with haemodynamic instability and abdominal pain. Investigation revealed haemorrhage from a lesion in his liver—an incidental finding of a hepatocellular carcinoma. Initial resuscitation and damage control surgery was performed at the peripheral hospital prior to transfer to a tertiary centre 386 km away for the second stage of management. The second stage of management included interventional radiological embolization of the bleeding liver vessel and subsequent resection of the liver tumour. This was all undertaken with new policies in place to limit the spread of infection at the peak of the COVID-19 epidemic.

## INTRODUCTION

Spontaneous haemorrhage of a liver tumour is a rare event with significant morbidity and mortality [1]. Hepatic adenoma and hepatocellular carcinoma (HCC) are the two most common associated pathologies [1]. HCC is the fourth most common cause of cancer-related death worldwide [2]. A spontaneous haemorrhage occurs in 3–26% of HCC cases with a 32–75% 30-day mortality, which may be attributed to atypical presentation compounded by poor overall condition [1, 3–5]. Factors associated with poor prognosis include poor liver reserve, advanced disease and severity of haemorrhage [5]. Patients on anti-coagulant pharmacotherapy do not have an increased risk of spontaneous haemorrhage or poorer prognosis [1].

## CASE REPORT

An 84-year-old male was brought in by ambulance to a rural hospital’s emergency department with acute epigastric pain associated with haemodynamic instability on a background of ischaemic heart disease, atrial fibrillation, hypertension and hypercholesterolaemia. His surgical history included coronary artery bypass grafting and the insertion of a dual chamber pacemaker. Regular pharmacotherapy included low-dose aspirin, rivaroxaban, sotolol and atorvastatin. He had no underlying liver disease or any significant risk factors for liver disease.

**Figure 1:**
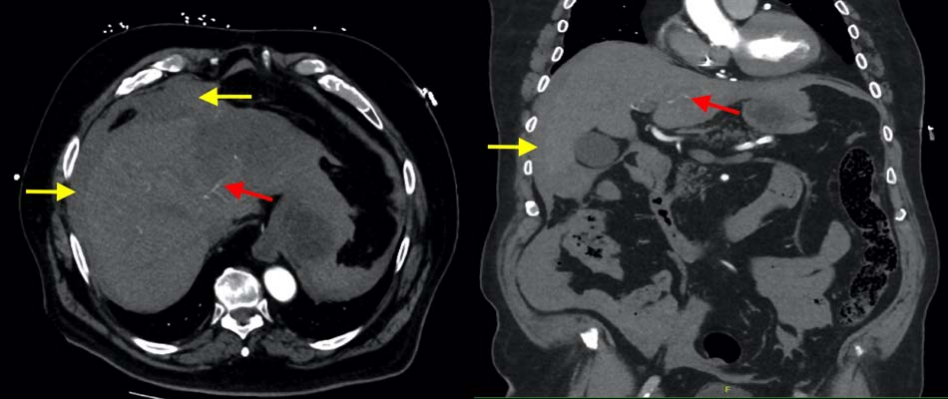
Axial and coronal contrast images of abdomen (yellow arrow—haemoperitoneum, red arrow—contrast extravasation from segment II/III artery).

He was hypotensive (systolic 60 mmHg) with a grossly distended abdomen that was tender to palpation in all quadrants with associated guarding and localized peritonism in the right upper quadrant. A bedside ultrasound of the abdomen suggested a peri-hepatic collection. After initial resuscitation, haemodynamics improved and the decision was made to perform a computer tomography (CT) angiogram to identify a bleeding source. This confirmed a peri-hepatic collection predominantly located around the left lobe of the liver with contrast extravasation from a prominent vessel extending towards the liver capsule (Fig. 1). Resuscitation continued with the activation of a massive transfusion protocol. Prothrombin concentrate and tranexamic acid were administered to partially reverse rivaroxaban, after coagulation studies revealed an international normalized ratio (INR) of 6.7. A Child-Pugh score of 9 (Class B) was calculated, placing the patient at a 30% risk of peri-operative mortality.

**Figure 2:**
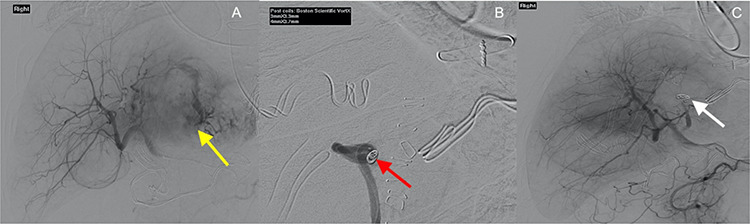
Interventional angiography of the liver (yellow arrow—extravasation of contrast, red arrow—coils placed in segment II/III artery and white arrow—coil in segment II/III artery with no evidence of contrast extravasation).

**Figure 3:**
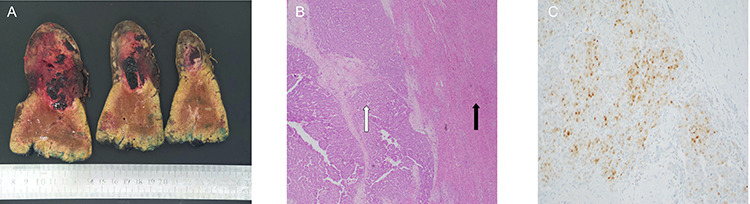
(**A**) macroscopic specimen demonstrating slices through a haemorrhagic lesion with normal liver parenchyma; (**B**) images demonstrating liver with intact architecture (black arrow) with HCC (white arrow) and (**C**) patchy nuclear staining with arginase-1 immunohistochemical stain.

The patient proceeded to an exploratory laparotomy. Induction of the patient followed COVID-19 guidelines by the Australian and New Zealand College of Anaesthetists, which suggested limiting theatre personnel to the anaesthetic team only, requiring personnel to wear N-95 face masks and intubating the patient under a clear plastic sheet. Once the patient was intubated, the surgical team re-entered the theatre and proceeded with a laparotomy. Laparotomy confirmed haemoperitoneum with active haemorrhage from a lesion in the segment II/III of the liver. The Pringle manoeuvre was applied for 15 min, allowing haemostasis to be achieved with the application of sutures in the liver to approximate the bleeding edges of the lesion and packing of the right upper quadrant. Haemodynamic stability was achieved allowing transfer to a tertiary centre for ongoing management.

On arrival to the tertiary centre, the patient was admitted to an intensive care unit for ongoing resuscitation. The patient subsequently underwent selective angioembolization of a segment II/III artery using polyvinyl alcohol (PVA) particles (250–350 μm) and four micro-coils (Fig. 2). Seventy-two hours after presentation, the patient underwent a re-look laparotomy with a left lateral sectionectomy of the liver to resect the identified lesion.

Histopathological assessment revealed an 85-mm moderately differentiated HCC with evidence of vascular invasion that was completely excised with a 20-mm clear margin [TNM Staging (AJCC 8th edition): pT2] (Fig. 3). The patient’s recovery post-procedure was routine with transfer back to the rural hospital 13 days post-liver resection.

From the rural hospital, the patient was referred to a rehabilitation hospital and subsequently discharged home a week later. At the time of discharge, the patient had a Child-Pugh score of 7 (Stage B) and was Stage A on the Barcelona Clinic Liver Cancer Staging. The patient was reviewed by a hepatologist 3 months after the liver resection. A triple phase CT was performed at the time, which did not show any residual liver lesion. Biochemistry revealed an elevated alpha-fetoprotein. The patient was commenced on sorafenib with a plan for review in 3 months.

## DISCUSSION

Resuscitation, diagnosis and intervention are the management goals of a spontaneous HCC haemorrhage [1, 3–5]. Resuscitation incorporates haemodynamic monitoring and regimen with infusion of blood products based on haemodynamic status and degree of anaemia. Correction of coagulopathies is crucial [1]. Diagnosis is aided by medical imaging. Ultrasonography can be performed at bedside in the unstable patient and may detect intra-peritoneal fluid, intra-hepatic haematoma or a liver lesion [1, 5]. In the stable patient, a triple phase CT of the abdomen can identify peritoneal collection, a bleeding source (if rate of bleeding is 1 mL/min) and/or a liver lesion [1, 5]. The immediate goal in intervention is to arrest haemorrhage. Selective angioembolization is currently the treatment of reference in stable or persistently unstable patients [1]. The associated morbidity is less than surgery, but decompensation of the underlying liver disease is a material risk [1]. Two options for emergency surgery are liver resection or local haemostatic procedures [1, 3]. The outcome of emergency liver resection is average with no survival benefit compared to patients undergoing resection at a later stage [1]. Emergency haemostatic procedures incorporate the principles of damage control surgery with definitive management performed at a later stage once the patient is resuscitated [1, 3, 5]. Advancement in resuscitation practices, imaging modalities and intervention have decreased the mortality associated with the spontaneous haemorrhage of a liver tumour [4, 5].

## CONFLICT OF INTEREST STATEMENT

None declared.

## FUNDING

None.
